# Association Between a Novel Adult Congenital Heart Disease Patient-Reported Outcome Metric and New York Heart Association Functional Classification

**DOI:** 10.1016/j.cjcpc.2026.04.002

**Published:** 2026-04-27

**Authors:** Rawan Amir, Jong Mi Ko, Shelby Kutty, Liesbet Van Bulck, Jamie L. Jackson, Maryanne Caruana, Susan M. Jameson, Vaikom S. Mahadevan, Philip Moons, Ari Cedars, APPROACH-IS II consortium, International Society for Adults Congenital Heart Disease (ISACHD)

**Affiliations:** a Division of Cardiology, Department of Medicine, Johns Hopkins Hospital School of Medicine, Baltimore, Maryland, USA; b Division of Pediatric Cardiology, Department of Pediatrics, Johns Hopkins School of Medicine, Baltimore, Maryland, USA; c Department of Public Health and Primary Care, KU Leuven—University of Leuven, Leuven, Belgium; d Center for Biobehavioral Health, Nationwide Children’s Hospital, Columbus, Ohio, USA; e Department of Cardiology, Mater Dei Hospital, Msida, Malta; f Department of Pediatrics and Medicine, Stanford University School of Medicine, Stanford, California, USA; g University of Massachusetts Chan School of Medicine, Worcester, Massachusetts, USA; h University of Gothenburg Centre for Person-Centred Care (GPCC), Sahlgrenska Academy, Gothenburg, Sweden; i Department of Paediatrics and Child Health, University of Cape Town, Cape Town, South Africa

**Keywords:** patient reported outcome metric, adult congenital heart disease, NYHA functional class

## Abstract

Patient-reported outcomes (PROs) are important for clinical care. As part of the Adults with Congenital Heart Disease International Study-II, we included an English language version of the novel adult congenital heart disease (ACHD)–specific PRO metric, the ACHD PRO, for distribution at English-speaking sites. We found that ACHD PRO scores correlate with New York Heart Association functional class, with an 11-point change in summary score reflecting a 1-category change in New York Heart Association class. These results provide further validation of the ACHD PRO as a tool and suggest clinically meaningful thresholds for differences in the ACHD PRO summary score.

## Résumé

Les résultats rapportés par les patients sont importants pour les soins cliniques. Dans le cadre de l’étude internationale II - Adults With Congenital Heart Disease (ACHD), nous avons inclus la version anglaise d’un nouvel outil de mesure des résultats rapportés par les patients portant sur les cardiopathies congénitales chez l'adulte (ACHD PRO) en vue de sa distribution dans des centres anglophones. Nous avons noté une corrélation entre les scores ACHD PRO et la classe fonctionnelle de la New York Heart Association, une variation de 11 points du score sommaire correspondant à une variation de 1 catégorie de la classification de la New York Heart Association. Ces résultats confirment la validité du score ACHD PRO en tant qu’outil d’évaluation et permettent de définir des seuils cliniquement significatifs pour les différences au niveau du score sommaire ACHD PRO.

As the adult congenital heart disease (ACHD) population grows and ages, the prevalence of heart failure in this group is increasing. It is estimated that 22% of ACHD admissions in the United States are secondary to heart failure.^1^ In the ACHD population, these admissions are associated with high rates of major adverse cardiovascular events, readmissions, and 1-year mortality.^1^

In the non-ACHD population, a variety of risk stratification tools have been used to assess clinical status with the goal of identifying patients at risk for clinical deterioration. Among the most common and prognostically informative is the New York Heart Association functional classification (NYHA FC). In the ACHD population, NYHA FC is not only predictive of outcomes but also closely linked with patient-reported quality of life (QoL).^2^, ^3^, ^4^

In the ACHD population, there is evidence that patient-reported limitations may not correlate with objective measurement.^5^ Nevertheless, patient-reported experiences remain a crucial element of clinical care as both an independent and prognostically informative outcome.^6^ The availability of a patient-reported health status metric that also accurately captures physician-assessed clinical status as measured by NYHA FC would, therefore, be a powerful metric for use in clinical care. In the present analysis, we investigated the association between scores on the ACHD disease-specific patient-reported outcome (PRO) metric, the ACHD PRO version 1 (V.1), and NYHA FC.^7^ Our data were derived from the international multicenter study—Assessment of Patterns of Patient-Reported Outcomes in Adults with Congenital Heart Disease International Study-II (APPROACH IS-II)—as a substudy for which the ACHD PRO V.1 was distributed to patients with ACHD diagnosed before age 10 at 6 centers in English-speaking countries. Further details regarding study design and methods have been previously published.^8^

A total of 424 patients were included in the present analysis. Median age in this cohort was 35 years, 86% were White, and 63% were female. Most patients had either moderate or severe American Heart Association heart defect complexity at 59% and 31%, respectively, and were American Heart Association physiologic stage B or C at 27% and 50%, respectively. Sixty-nine percent of patients reported NYHA class I symptoms, 26% class II, and 5% class III. When comparing the ACHD PRO V.1 summary score with NYHA FC, we found that a change of 11.4 points was associated with a one-class difference. Separated into its specific domains, there was a general association between lower ACHD PRO V.1 domain scores and lower NYHA class (Table 1). This association was strongest in the physical limitations domain (–13.9 ACHD PRO V.1 points per NYHA class; *P* < 0.001) and the QoL domain (–13.6; *P* < 0.001). Changes in scores relative to NYHA class were similar among the anxiety/depression, symptoms, and arrhythmia domains (–10.5, –10.2, and –9.1, respectively), all achieving statistical significance (*P* < 0.001).

**Table 1:** NYHA FC and ACHD PRO V.1 domain scores

Score domains	NYHA I (reference) score (95% CI)	NYHA II effect difference (95% CI, *P* value)	NYHA III effect difference (95% CI, *P* value)
Composite	87 (85-89)	–11 (–14 to –7, <0.001)	–24 (–30 to –18, <0.001)
Symptoms	85 (83-87)	–10 (–13 to –7, <0.001)	–20 (–27 to –13, <0.001)
Physical limitation	86 (84-88)	–11 (–15 to –7, <0.001)	–33 (–41 to –25, <0.001)
Arrhythmia	88 (86-90)	–9 (–13 to –5, <0.001)	–18 (–26 to –11, <0.001)
Quality of life	91 (89-93)	–12 (–16 to –8, <0.001)	–29 (–37 to –21, <0.001)
Anxiety/depression	86 (84-88)	–10 (–14 to –7, <0.001)	–21 (–29 to –13, <0.001)

ACHD PRO, adult congenital heart disease patient-reported outcome; CI, confidence interval; NYHA FC, New York Heart Association Functional Class.

These findings provide further data validating the ACHD PRO V.1 as an outcome metric. NYHA FC, as expected, had the greatest association with the physical limitations domain of the ACHD PRO V.1, supporting the content validity of this domain. The observed relationship between NYHA FC and the QoL domain further supports this conclusion, given existing data from the APPROACH-IS study, suggesting a strong relationship between QoL and NYHA FC in the ACHD population.^8^ Most importantly, these findings support the use of the ACHD PRO V.1 summary score as a potentially valuable patient-centered tool for early detection of clinical deterioration and disease progression.

Despite these promising results, one must recognize the limitations of these data. Data were derived in a retrospective manner, increasing the risk of nonresponse, recall bias, and social desirability bias. Causality cannot be determined by a study of such design. Most importantly, the present data are in a limited English-speaking cohort of predominantly North American academic medical centers, and the generalizability of the present findings outside of this setting is therefore not certain. More data should be available from international sites in upcoming years as part of the Congenital Outcomes and Reported Experience in ACHD (CORE-ACHD) initiative.

Although larger scale studies are needed, these findings further support the validity of the ACHD PRO V.1 questionnaire, revealing a clear association between physician-determined NYHA FC and PROs. They also suggest a summary score difference of 11 points on the ACHD PRO V.1 as a potentially clinically meaningful threshold. Future prospective studies will need to investigate the degree to which the ACHD PRO V.1 is both sensitive to changes in clinical status and predictive of major adverse cardiac events and hard clinical outcomes.

## Ethics Statement

The work presented in this manuscript complied with ethical guidelines for conduct of human research and was approved by the local institutional review boards at all participating institutions.

## Patient Consent

The authors confirm that patient consent forms have been obtained for this article.

## Funding Sources

The authors have no funding sources to declare.

## Disclosures

The authors have no conflicts of interest to disclose.
